# Supplementary material for: Enhanced streamflow prediction with SWAT using support vector regression for spatial calibration: A case study in the Illinois River watershed, U.S
Source: PLoS One. 2021 Apr 12;16(4):e0248489. doi: 10.1371/journal.pone.0248489 (PMC8041176; doi:10.1371/journal.pone.0248489)
Supplement: S1 Appendix — (DOCX) [file pone.0248489.s001.docx]

**S1 Appendix.**

After parameter sensitivity analysis, we selected 13 and 10 SWAT parameters for the wet and dry season, respectively. Then, we used these parameters to conduct the calibration and validation in SWAT-CUP.

**S1 Table. The initial parameters and their range in calibration by SWAT-CUP. Two season calibration of SWAT was used to improve and better capture the seasonal dynamics of the watershed system.**

| No | Parameter Name^1^ | Parameter Description | Range | Season | |
| --- | --- | --- | --- | --- | --- |
|  |  |  |  | **If used in the wet season** | **If used in the dry season** |
| 1 | R__CN2.mgt | SCS runoff curve number II | -0.25-0.25 | Yes | Yes |
| 2 | V__ALPHA_BF.gw | Baseflow alpha factor (1 day^−1^) | 0–1 | Yes | Yes |
| 3 | V__GWQMN.gw | Threshold depth of water in the shallow aquifer required for return flow to occur (mm H_2_O) | 0–2000 | Yes | Yes |
| 4 | V__GW_REVAP.gw | Groundwater “revap” coefficient | 0.02–0.2 | Yes | Yes |
| 5 | V__EPCO.hru | Plant uptake compensation factor | 0–1 | Yes | Yes |
| 6 | R__SOL_K (1).sol | Saturated hydraulic conductivity at the 1st soil layer (mm h^−1^) | 30-102 | Yes | Yes |
| 7 | R__SOL_AWC (1).sol | Available water capacity of the 1st soil layer (mm H_2_O mm soil^−1^) | 0.08-0.2 | Yes | No |
| 8 | R__SOL_BD (1).sol | Moist bulk density at the 1st soil layer (g cm^−3^) | 1.3-1.45 | Yes | No |
| 9 | A__OV_N.hru | Manning’s “n” value for overland flow | 0.01–30 | Yes | No |
| 10 | A__CH_K2.rte | Effective hydraulic conductivity in main channel alluvium (mm h^−1^) | −0.01–500 | Yes | Yes |
| 11 | R__HRU_SLP.hru | Average slope steepness (m m^−1^) | 0–1 | Yes | Yes |
| 12 | V_RCHRG_DP.gw | Deep aquifer percolation fraction | 0-1 | Yes | Yes |
| 13 | A_CH_K1 | Effective hydraulic conductivity in tributary channel alluvium | 0-300 | Yes | No |
| 14 | V_ESCO.hru | Soil evaporation compensation factor | 0-1 | No | Yes |

^1^ Note: “*A*__”, “*V*__” and “*R*__” mean an absolute increase, a replacement, and a relative change to the initial parameter values in SWAT-CUP, respectively.
