# Supplementary material for: Enhanced streamflow prediction with SWAT using support vector regression for spatial calibration: A case study in the Illinois River watershed, U.S
Source: PLoS One. 2021 Apr 12;16(4):e0248489. doi: 10.1371/journal.pone.0248489 (PMC8041176; doi:10.1371/journal.pone.0248489)
Supplement: Attachment — Submitted filename: Response to Reviewers.docx [file pone.0248489.s002.docx]

PLOS ONE Academic Editor Mou Leong Tan,

Thank you for the excellent comments and opportunity to resubmit our manuscript. We have made all the changes and provided direct responses to each comment. We have added an appendix and several editorial changes. We have addressed the copyright issue in our figure 1 and produced a new figure based on publicly available data and licensed software. We have uploaded our data to *doi.org/10.23719/1520734* which will be available upon acceptance and publication.

Please accept our submission to Plos One Hydrology on the evaluation of an approach to improve streamflow prediction by combining a machine learning method (i.e., support vector regression) and the Soil and Water Assessment Tool (SWAT).

This research highlights three points: 1. An integrated SWAT model and SVR can raise the prediction accuracy of streamflow simulation with the ability to capture some intrinsic non-linear behaviors between rainfall and runoff while respecting the mechanisms of runoff generation and may serve as a reliable regional tool for an ungauged or limited data watersheds; 2. We found that wet and dry seasons showed a different response to prediction improvement likely based on different underlying intrinsic controls of discharge which provides insight to when and why some machine learning approaches are useful; 3. By applying our hybrid method at thirteen hydrology stations located the Illinois River Watershed in the U.S. our manuscript shows how machine learning techniques can be integrated into conventional calibration and validation approaches to improve prediction and capture complexity of input data that are otherwise would be lost.

Thank you again for the opportunity,

Ken Forshay

Lifeng Yuan

Response to editor and reviewer comments. Italicized text is our response.

Editor Comments:

Journal Requirements:

When submitting your revision, we need you to address these additional requirements.

1. Please ensure that your manuscript meets PLOS ONE's style requirements, including those for file naming. The PLOS ONE style templates can be found at

<https://journals.plos.org/plosone/s/file?id=wjVg/PLOSOne_formatting_sample_main_body.pdf> and

<https://journals.plos.org/plosone/s/file?id=ba62/PLOSOne_formatting_sample_title_authors_affiliations.pdf>

***Answer:*** *We appreciate the Academic Editor’s concern on the format of our initial manuscript.* *We have formatted our manuscript according to above two template files.* *Please let us know if any aspect of our manuscript does not follow the format requirement of the PloS One journal.*

2. In your Data Availability statement, you have not specified where the minimal data set underlying the results described in your manuscript can be found. PLOS defines a study's minimal data set as the underlying data used to reach the conclusions drawn in the manuscript and any additional data required to replicate the reported study findings in their entirety. All PLOS journals require that the minimal data set be made fully available. For more information about our data policy, please see <http://journals.plos.org/plosone/s/data-availability>.

Upon re-submitting your revised manuscript, please upload your study’s minimal underlying data set as either Supporting Information files or to a stable, public repository and include the relevant URLs, DOIs, or accession numbers within your revised cover letter. For a list of acceptable repositories, please see <http://journals.plos.org/plosone/s/data-availability#loc-recommended-repositories>. Any potentially identifying patient information must be fully anonymized.

Important: If there are ethical or legal restrictions to sharing your data publicly, please explain these restrictions in detail. Please see our guidelines for more information on what we consider unacceptable restrictions to publicly sharing data: <http://journals.plos.org/plosone/s/data-availability#loc-unacceptable-data-access-restrictions>. Note that it is not acceptable for the authors to be the sole named individuals responsible for ensuring data access.

We will update your Data Availability statement to reflect the information you provide in your cover letter.

***Answer****: Yes, we have uploaded the study data in a stable, public repository* *as DOI: 10.23719/1520734 . Anyone can freely access our study data at doi.org/10.23719/1520734 upon publication.*

*The upload data are divided into two groups: spatial data and time-series data. Spatial data are Digital elevation model (DEM) that obtained from Shuttle Radar Topography Mission (SRTM) 1 Arc-Second (about 30 m × 30 m) Global Database and downloaded from USGS website (*[*https://earthexplorer.usgs.gov/*](https://earthexplorer.usgs.gov/)*, 01-28-2018) (Fig 2a). Land use and land cover (LULC) data was from the 2011 NLCD dataset (*[*https://www.mrlc.gov/*](https://www.mrlc.gov/nlcd11_data.php)*, 01-31-2018) (Fig 2b), and spatial resolution is 100 m × 100 m. Soil data came from the SSURGO database (*[*https://websoilsurvey.nrcs.usda.gov/*](https://websoilsurvey.nrcs.usda.gov/)*, 02-05-2018) (Fig 2c). Time-series data include climate and discharge data. Climate data obtained from the National Climatic Data Center (NCDC) (*[*https://www.ncdc.noaa.gov*](https://www.ncdc.noaa.gov)*/, 02-07-2018) (Fig 2d). Due to missing precipitation and temperature records from NCDC climate data from Jan. 1990 to Dec. 2013, we downloaded alternative Climate Forecast System Reanalysis (CFSR) data from the SWAT official website (*[*https://globalweather.tamu.edu/*](https://globalweather.tamu.edu/)*, 01-31-2018), then filled missing NCDC data using climate data from the closest CFSR grid stations (not shown in Fig 2d). The streamflow data came from thirteen U.S. Geological Survey (USGS) hydrologic stations. These monthly discharge data can be accessed and downloaded by USGS official website (*[*https://dashboard.waterdata.usgs.gov/app/nwd/?region=lower48*](https://dashboard.waterdata.usgs.gov/app/nwd/?region=lower48)*).*

3. We note that Figures 1, 2 and 5 in your submission contain [ap/satellite images which may be copyrighted.

All PLOS content is published under the Creative Commons Attribution License (CC BY 4.0), which means that the manuscript, images, and Supporting Information files will be freely available online, and any third party is permitted to access, download, copy, distribute, and use these materials in any way, even commercially, with proper attribution. For these reasons, we cannot publish previously copyrighted maps or satellite images created using proprietary data, such as Google software (Google Maps, Street View, and Earth). For more information, see our copyright guidelines: <http://journals.plos.org/plosone/s/licenses-and-copyright>.

We require you to either (a) present written permission from the copyright holder to publish these figures specifically under the CC BY 4.0 license, or (b) remove the figures from your submission:

a. You may seek permission from the original copyright holder of Figures 1, 2 and 5 to publish the content specifically under the CC BY 4.0 license.

We recommend that you contact the original copyright holder with the Content Permission Form (http://journals.plos.org/plosone/s/file?id=7c09/content-permission-form.pdf) and the following text:

“I request permission for the open-access journal PLOS ONE to publish XXX under the Creative Commons Attribution License (CCAL) CC BY 4.0 (http://creativecommons.org/licenses/by/4.0/). Please be aware that this license allows unrestricted use and distribution, even commercially, by third parties. Please reply and provide explicit written permission to publish XXX under a CC BY license and complete the attached form.”

Please upload the completed Content Permission Form or other proof of granted permissions as an "Other" file with your submission.

In the figure caption of the copyrighted figure, please include the following text: “Reprinted from [ref] under a CC BY license, with permission from [name of publisher], original copyright [original copyright year].”

b. If you are unable to obtain permission from the original copyright holder to publish these figures under the CC BY 4.0 license or if the copyright holder’s requirements are incompatible with the CC BY 4.0 license, please either i) remove the figure or ii) supply a replacement figure that complies with the CC BY 4.0 license. Please check copyright information on all replacement figures and update the figure caption with source information. If applicable, please specify in the figure caption text when a figure is similar but not identical to the original image and is therefore for illustrative purposes only.

The following resources for replacing copyrighted map figures may be helpful:

USGS National Map Viewer (public domain): http://viewer.nationalmap.gov/viewer/

The Gateway to Astronaut Photography of Earth (public domain): http://eol.jsc.nasa.gov/sseop/clickmap/

Maps at the CIA (public domain): https://www.cia.gov/library/publications/the-world-factbook/index.html and https://www.cia.gov/library/publications/cia-maps-publications/index.html

NASA Earth Observatory (public domain): http://earthobservatory.nasa.gov/

Landsat: <http://landsat.visibleearth.nasa.gov/>

USGS EROS (Earth Resources Observatory and Science (EROS) Center) (public domain): http://eros.usgs.gov/#

Natural Earth (public domain): http://www.naturalearthdata.com/

**Answer**: *We have deleted the google map in Figure 1 within our manuscript to follow the requirement of publication. We used ESRI ArcGIS software to generate Figure 1, 2, and 8, and applied Microsoft Visio to generate Figure 5. And, EPA possesses the using licensed versions of ESRI ArcGIS and Microsoft Visio.*

*All data but the google map in the previous Figure 1 used in our research are derived from public data from public domains and produced by the authors. Anyone can download these data from the corresponding public repository listed.*

4. Please include your tables as part of your main manuscript and remove the individual files. Please note that supplementary tables should remain as separate "supporting information" files.

***Answer****: We have added the tables into the main body of our manuscript and removed the table document in the submitting system.*

Reviewers' comments:

Reviewer's Responses to Questions

**Comments to the Author**

1. Is the manuscript technically sound, and do the data support the conclusions?

The manuscript must describe a technically sound piece of scientific research with data that supports the conclusions. Experiments must have been conducted rigorously, with appropriate controls, replication, and sample sizes. The conclusions must be drawn appropriately based on the data presented.

Reviewer #1: Partly

Reviewer #2: Yes

Reviewer #3: Partly

2. Has the statistical analysis been performed appropriately and rigorously?

Reviewer #1: Yes

Reviewer #2: Yes

Reviewer #3: I Don't Know

3. Have the authors made all data underlying the findings in their manuscript fully available?

The [PLOS Data policy](https://gcc01.safelinks.protection.outlook.com/?url=http%3A%2F%2Fwww.plosone.org%2Fstatic%2Fpolicies.action%23sharing&data=04%7C01%7CForshay.Ken%40epa.gov%7C4a4307e3f4a648dffa1508d8915b6184%7C88b378b367484867acf976aacbeca6a7%7C0%7C0%7C637419168959521506%7CUnknown%7CTWFpbGZsb3d8eyJWIjoiMC4wLjAwMDAiLCJQIjoiV2luMzIiLCJBTiI6Ik1haWwiLCJXVCI6Mn0%3D%7C2000&sdata=hxq84w7hpvrk41Qr7GOaKbiTIGLcqV%2FxhwrIayb220c%3D&reserved=0) requires authors to make all data underlying the findings described in their manuscript fully available without restriction, with rare exception (please refer to the Data Availability Statement in the manuscript PDF file). The data should be provided as part of the manuscript or its supporting information or deposited to a public repository. For example, in addition to summary statistics, the data points behind means, medians and variance measures should be available. If there are restrictions on publicly sharing data—e.g. participant privacy or use of data from a third party—those must be specified.

Reviewer #1: No

Reviewer #2: Yes

Reviewer #3: Yes

4. Is the manuscript presented in an intelligible fashion and written in standard English?

PLOS ONE does not copyedit accepted manuscripts, so the language in submitted articles must be clear, correct, and unambiguous. Any typographical or grammatical errors should be corrected at revision, so please note any specific errors here.

Reviewer #1: Yes

Reviewer #2: Yes

Reviewer #3: Yes

5. Review Comments to the Author

Please use the space provided to explain your answers to the questions above. You may also include additional comments for the author, including concerns about dual publication, research ethics, or publication ethics. (Please upload your review as an attachment if it exceeds 20,000 characters)

Reviewer #1: This study proposed a method to improve monthly streamflow prediction performance by coupling a seasonal Support Vector Regression (SVR) model with the Soil and Water Assessment Tool (SWAT) model, and applied it in the Illinois River watershed (IRW), U.S. Overall, this paper presents an interesting approach for improving streamflow predictions. However, I think the following issues should be addressed before the paper is considered for publication.
1) I do not understand why the authors chose the approach to calibrate and validate the SWAT-VAR model by leaving out one station. This means that the authors need to develop 13 SWAT-SVR models, whose final parameter values could be rather different (unfortunately, the authors did not discuss this point in the paper). In this case, what should be the SWAT-SVR model for the entire watershed? In my opinion, the traditional approach that includes all stations but divides the study period into the calibration and validation periods works better here.

***Answer:*** *In the revision on line 416 we have cited and described our approach. We appreciate reviewer’s concern on the technical detail of our manuscript. Here we used a spatial proxy approach. Generally, conventional calibration method of SWAT or other watershed models could be roughly divided into two groups: temporal and spatial calibrations. Temporal calibration is typically performed by splitting the available observed data into two datasets according to different periods: one for calibration, and another for validation. However, data may also be split spatially, with all available data at a given measured location assigned to the calibration phase and correspondingly perform the validation at one or more other gauges within the watershed. This method is spatial calibration (or also named spatial proxy basin approach). This spatial calibration approach is useful when users are faced with data-limited, ungauged situations or a study area is a large watershed. We present an approach and method that may be ultimately applied to an ungauged watershed. As Prasad Daggupati et al. (2015) pointed out, a regular temporal calibration method may not work well for a large size watershed due to potential spatial variability within the basin. Our study area is over 4,200 km^2^, and available data length from 13 USGS hydrologic station are not consistent (07196090 and 07196973 stations have only 42 and 96 data point in this study). Use of a temporal method to divide the datasets for calibration (say 70%) and validation subset (30%) due to a lack of similar numbers of wet, moderate, and dry years occurring in both periods may not be appropriate (Gan et al., 1997) albeit more familiar.*

*Spatial calibration and validation approaches have been performed in several previous SWAT studies (e.g. Arnold et al.,2001, Van Liew and Garbrecht, 2003, Cao et al., 2006, Daggupati et al., 2015). Leave-one-out is a particular kind of spatial calibration method we used for this manuscript. For example, Navideh Noori et al. (2016) applied the leave-one-out method to develop a hybrid SWAT coupling ANN method.*

*We did develop 26 SWAT-SVR models (for wet and dry seasons) and corresponding 26 SWAT models calibrated by SWAT-CUP. In 26 SWAT-SVR models, we used SWAT parameters by default (no calibration). For the SVR approach, 3 parameters (C, γ, ε) needed to be determined. The final value range of C for 26 SWAT-SVR models are from 53.0156 to 255.0156, the value of 𝛾 is 0.4, and ɛ is 0.00390625 for the wet season; the value of C falls between 32.0156 and 255.0156, 𝛾 is 1.2, and ɛ is 0.00390625 as well in the dry season. We can see C value have a wide range of change, but 𝛾 and ɛ are similar during the wet and dry seasons, respectively. This is because for any two runs there are always 11 common watersheds in SWAT-SVR. We made Fig. 5 (research flowchart) to help readers better understand our research processes.*

2) The SWAT model is a continuous simulation model. I could not fathom how the authors could run SWAT-CUP for dry and wet seasons independently. The authors have not provided any SWAT model parameter calibration results in the paper.

***Answer:*** *We have added an appendix e.g. Table 1 to help better describe our approach. We agree with reviewer’s opinion that SWAT model is a physically-based, continuous watershed model. However, that does not mean we cannot calibrate SWAT by dividing dry and wet seasons in SWAT-CUP. In fact, SWAT assumes that model parameters are season insensitive and attempt to identify ‘optimal’ values that would describe watershed behavior during dry and wet seasons. We considered and evaluated continuous unseparated calibration, but this assumption would compromise accuracy of model predictions. As we know, hydrologic models often perform poorly in simulation dry years in areas with large inter-annual variability in precipitation since the temporal variations in model parameters which exist in watersheds are not considered. Another reason is related with the objective function such as R^2^ and NSE, which can better reflect the hydrologic characteristics in wet periods than ones in dry periods. Misgana K. Muleta (2012), Dejian Zhang (2015), and Xin Gao(2018) published their paper which discussed in detail the method improvement of SWAT calibration by separating wet and dry seasons.*

*Technically, it is straightforward to separate dry and wet season in SWAT-CUP because this software supports the leap-day, month, or year calibration. For example, the calibration file of separated wet and dry seasons in SWAT-CUP could be written as below:*

| *No.* | *Wet season* | *Obs.* | *No.* | *Dry season* | *Obs.* |
| --- | --- | --- | --- | --- | --- |
| *3* | *FLOW_OUT_3_1995* | *0.89* | *1* | *FLOW_OUT_1_1995* | *1.04* |
| *4* | *FLOW_OUT_4_1995* | *0.89* | *2* | *FLOW_OUT_2_1995* | *0.69* |
| *5* | *FLOW_OUT_5_1995* | *1.20* | *6* | *FLOW_OUT_6_1995* | *1.22* |
| *9* | *FLOW_OUT_9_1995* | *0.16* | *7* | *FLOW_OUT_7_1995* | *0.85* |
| *10* | *FLOW_OUT_10_1995* | *0.11* | *8* | *FLOW_OUT_8_1995* | *0.33* |
| *11* | *FLOW_OUT_11_1995* | *0.10* | *12* | *FLOW_OUT_12_1995* | *0.34* |
| *15* | *FLOW_OUT_3_1996* | *0.27* | *13* | *FLOW_OUT_1_1996* | *0.57* |
| *16* | *FLOW_OUT_4_1996* | *0.44* | *14* | *FLOW_OUT_2_1996* | *0.34* |
| *17* | *FLOW_OUT_5_1996* | *0.41* | *18* | *FLOW_OUT_6_1996* | *0.44* |
| *21* | *FLOW_OUT_9_1996* | *0.69* | *19* | *FLOW_OUT_7_1996* | *0.11* |
| *22* | *FLOW_OUT_10_1996* | *0.37* | *20* | *FLOW_OUT_8_1996* | *0.03* |
| *23* | *FLOW_OUT_11_1996* | *1.92* | *24* | *FLOW_OUT_12_1996* | *1.07* |
| *…* | *…* | *…* | *…* | *…* | *…* |

*Note that these observations must be ranked by time series order in SWAT-CUP to run successfully.*

*In addition, we added the SWAT parameters initial range at SWAT-CUP calibration as below:*

**Table 1. The initial parameters and their range in calibration.**

| No | Parameter Name^1^ | Parameter Description | Range | Season | |
| --- | --- | --- | --- | --- | --- |
|  |  |  |  | **If used in the wet season** | **If used in the dry season** |
| 1 | R__CN2.mgt | SCS runoff curve number II | -0.25-0.25 | Yes | Yes |
| 2 | V__ALPHA_BF.gw | Baseflow alpha factor (1 day^−1^) | 0–1 | Yes | Yes |
| 3 | V__GWQMN.gw | Threshold depth of water in the shallow aquifer required for return flow to occur (mm H_2_O) | 0–2000 | Yes | Yes |
| 4 | V__GW_REVAP.gw | Groundwater “revap” coefficient | 0.02–0.2 | Yes | Yes |
| 5 | V__EPCO.hru | Plant uptake compensation factor | 0–1 | Yes | Yes |
| 6 | R__SOL_K (1).sol | Saturated hydraulic conductivity at the 1st soil layer (mm h^−1^) | 30-102 | Yes | Yes |
| 7 | R__SOL_AWC (1).sol | Available water capacity of the 1st soil layer (mm H_2_O mm soil^−1^) | 0.08-0.2 | Yes | No |
| 8 | R__SOL_BD (1).sol | Moist bulk density at the 1st soil layer (g cm^−3^) | 1.3-1.45 | Yes | No |
| 9 | A__OV_N.hru | Manning’s “n” value for overland flow | 0.01–30 | Yes | No |
| 10 | A__CH_K2.rte | Effective hydraulic conductivity in main channel alluvium (mm h^−1^) | −0.01–500 | Yes | Yes |
| 11 | R__HRU_SLP.hru | Average slope steepness (m m^−1^) | 0–1 | Yes | Yes |
| 12 | V_RCHRG_DP.gw | Deep aquifer percolation fraction | 0-1 | Yes | Yes |
| 13 | A_CH_K1 | Effective hydraulic conductivity in tributary channel alluvium | 0-300 | Yes | No |
| 14 | V_ESCO.hru | Soil evaporation compensation factor | 0-1 | No | Yes |

^1^ Note: “*A*__”, “*V*__” and “*R*__” mean an absolute increase, a replacement, and a relative change to the initial parameter values, respectively.

3) There are some logical flaws in the authors’ discussions related to Fig. 9. What is presented in Fig 9 is the evaluation statistics solely for the validated watershed. However, each SWAT-SVR model was developed using the data of the other 12 watersheds of various sizes. Performance at the single validated watershed is not sufficient to judge the model’s overall performance, let alone, to determine the application scope of the SWAT-SVR model. This judgement should be based on the model performance at all 13 watersheds. This is why I suggest the authors drop the “leaving-out-one-watershed” approach for calibration and validation.

***Answer:*** *We developed 26 SWAT-SVR models for different seasons to represent the corresponding subwatersheds and verify the effectiveness of our hybrid method, not only for one or two models. We are thankful for the constructive comments from the reviewer and agree with the reviewer’s opinion that the single validated watershed is not enough to judge the model’s overall performance. This study attempts to improve the monthly streamflow at a large watershed by coupling SWAT and the SVR method. Spatial heterogeneity and temporal variability are intrinsically connected to watershed characteristics and in this system, we believe that segregating wet and dry season helps better describe the intrinsic dynamics of watershed hydrology, at least in this system. In this study, we developed 26 models for different subwatersheds. We don’t believe that a single continuous everywhere model is appropriate for such large basin given the seasonal variation and the likelihood that there are important changes to drivers of hydrology in the wet and dry seasons.*
4) The authors did not give any reason of including watershed area, but no other variable, in the SWAT-SVR model. Is it sufficient to include this single variable besides SWAT streamflow results in the model?

***Answer****: See lines 240-245. Our goal was to develop a model with few variable inputs .We appreciate reviewer’s concern on the model parameter selection of SWAT-SVR. We worked to develop an innovative method (a hybrid model) to improve the prediction of monthly streamflow in a large watershed. Since SVR is effectively a black-box model, we don’t consider all the physical parameters mechanistically that are interacting in potentially non-linear or chaotic ways inside the model, although SWAT does much of this, we wish to avoid more model variables or more complex mechanistic interactions that may not behave in a predictable way so that our SVR approach will better improve the model overall simulation. At first, we hoped to only use the streamflow from SWAT with default parameters as the sole input for SVR, but performance greatly improved when we added the variable -upstream drainage area into the simulations. We believe the upstream drainage area is the important parameter for this empirical mode because it plays a critical role at the early period of the hydrologic model, we describe the parameters on lines 233-239. Other variables such as terrain, soil, land use and land cover, weather, and others are included by SWAT. Here, we regarded the SWAT model essentially as a transfer function then hybridize it to improve prediction.*

Reviewer #2: 1) The parameters considered in SWAT calibration and SWAT-SVR Calibration are not discussed. Is both of the calibration parameters chosen are the same for both model?

***Answer:*** *We have added a table 1 in the appendix to better describe the model parameters.* *We agreed with reviewer’s opinion that the selection, sensitivity analysis and determination of SWAT parameters are very important for building a SWAT model. In our study, SWAT was applied with default values of parameters to simulate the streamflow at first, then we took the streamflow output from SWAT (no calibration) and upstream drainage area as input variables into the SVR model. For SVR, only three parameters need to be determined, and they are C, 𝛾, and ε which were determine by grid search and cross-validation. For SWAT-CUP, after parameter sensitivity analysis, we selected 13 parameters for wet season and 10 parameters for dry season, and applied the leave-one-out method and conducted spatial calibration for 13 stations. We attached initial parameters value range of SWAT-CUP before calibration for the wet and dry season. The ultimate parameter value or range of calibration for 13 stations are different one another because of the application of the spatial calibration method, so we don’t show all validation results from 13 stations in the manuscript. We focused on the model performance comparison of SWAT-SVR and SWAT-CUP.*

**Table 1. The initial parameters and their range in calibration.**

| No | Parameter Name^1^ | Parameter Description | Range | Season | |
| --- | --- | --- | --- | --- | --- |
|  |  |  |  | **If used in the wet season** | **If used in the dry season** |
| 1 | R__CN2.mgt | SCS runoff curve number II | -0.25-0.25 | Yes | Yes |
| 2 | V__ALPHA_BF.gw | Baseflow alpha factor (1 day^−1^) | 0–1 | Yes | Yes |
| 3 | V__GWQMN.gw | Threshold depth of water in the shallow aquifer required for return flow to occur (mm H_2_O) | 0–2000 | Yes | Yes |
| 4 | V__GW_REVAP.gw | Groundwater “revap” coefficient | 0.02–0.2 | Yes | Yes |
| 5 | V__EPCO.hru | Plant uptake compensation factor | 0–1 | Yes | Yes |
| 6 | R__SOL_K (1).sol | Saturated hydraulic conductivity at the 1st soil layer (mm h^−1^) | 30-102 | Yes | Yes |
| 7 | R__SOL_AWC (1).sol | Available water capacity of the 1st soil layer (mm H_2_O mm soil^−1^) | 0.08-0.2 | Yes | No |
| 8 | R__SOL_BD (1).sol | Moist bulk density at the 1st soil layer (g cm^−3^) | 1.3-1.45 | Yes | No |
| 9 | A__OV_N.hru | Manning’s “n” value for overland flow | 0.01–30 | Yes | No |
| 10 | A__CH_K2.rte | Effective hydraulic conductivity in main channel alluvium (mm h^−1^) | −0.01–500 | Yes | Yes |
| 11 | R__HRU_SLP.hru | Average slope steepness (m m^−1^) | 0–1 | Yes | Yes |
| 12 | V_RCHRG_DP.gw | Deep aquifer percolation fraction | 0-1 | Yes | Yes |
| 13 | A_CH_K1 | Effective hydraulic conductivity in tributary channel alluvium | 0-300 | Yes | No |
| 14 | V_ESCO.hru | Soil evaporation compensation factor | 0-1 | No | Yes |

^1^ Note: “*A*__”, “*V*__” and “*R*__” mean an absolute increase, a replacement, and a relative change to the initial parameter values, respectively.

2) It is mentioned in the paper that SUFI 2 is being use for SWAT calibration, however for SWAT-SVR Calibration, how is it being conducted?

***Answer****: This is described on line 232-240. We first divided wet and dry season subsets for each station (total is 13). For dry or wet dataset, we applied leave-one-out method to determine the calibration and validation dataset. For each calibration dataset, we used grid search and k-fold cross-validation method to optimize the SVR parameters (C,γ, ε) by defining the upper and lower bound for each parameter and estimating the predicted accuracy of the model. In the k-fold cross-validation, the dataset was subdivided into k subsets of nearly equal size. In each step, the k-1 subsets were used to train the model while the remaining subset was used for validation. After found the optimized parameter set for each station, we can calculate the evaluation indicator for calibration, and applied this parameter set to validation dataset and obtained the statistics for the validation period.*

3) Author's use 5 statistical approaches to identify the model accuracy, however based on Table 3, only 3 statistical approaches rating has been shown, it will be better to include another 2 statistics.

**Answer:** *We have added a citation to the table to represent that the table represents the more common statistical approaches used in SWAT calibration. In our study we indeed used 5 but adding the two parameters would be confusing for this table. We appreciate reviewer’s concern on how to evaluate the model performance. Table 3 was developed by Moriasi et al.,(2007) based on hundreds of studies. We believe the model performance will be easier to compare if we use this more common criterion to rate the model performance, but we also believe the added statistical approaches are also useful. However, we only followed the guideline developed by Moriasi et al., but didn’t develop further rating guideline for the additional statistics, which is beyond the scope of this paper.*

4) Is it a bit unclear on how the SWAT-SVR being programmed, is it via Mathlab? The author may want to elaborate more on the system.

**Answer:** *The approach is described on line 263. We appreciate reviewer’s interest in the technical details of our programming. We didn’t use the Matlab platform. Instead, we used R and ‘e1071’ package to code the script for building the SVR model for different seasons at 13 stations. We used ‘tune’ function to conduct grid-search and cross-validate for the parameter set of C, γ, and ε, and used ‘svm’ function to build SVR model based on RBF kernel function. In this manuscript, we concentrate on the comparison of two different methods, results analysis and discussion parts, so some subtle technical details were neglected. We don’t mind supplying the original R script of SWAT-SVR model development if that is necessary for publication.*

5) Figure 10 shows some high peak rainfall are unable to capture via both model, elaboration on this will help future researcher to consider the factors.

**Answer:** *Thank you, we have appended the discussion into the revision on Figure 10 and lines 453-455. High peak streamflow cannot be captured well, partly reason might be that it is difficult to capture measured flow value during flooding by the flow sensor. And, the purpose of SWAT development is not for flood prediction, which has been explained at SWAT user manual. Also, the performance of SWAT-SVR heavily relied on the training data, it did not perform well when predicting high flows due to a small amount of training data in this study.*

6) Overall the paper is a good paper with some good analysis and explanation and may help future researcher to conduct research on hydrological model.

**Answer: Thank you!** *We are thankful for reviewer’s comments. We also feel confident that our approach as well as the results and conclusions are useful.*

Reviewer #3: The present work “Enhanced Streamflow Prediction with SWAT Using Support Vector Regression for Spatial Calibration: A Case Study in the Illinois River Watershed, U.S.” is interesting and original. Its main point of interest and originality is the development of a hybrid SWAT and Support Vector Regression (SVR) model based on 13 hydrologic gauging stations in Illinois River, US
However, there are some points that need clarification or re-consideration by the authors.
Introductions:
1. On page 4, line 68-69, the authors argue that several studies in the past have evaluated the performance of SWAT and SVM models in streamflow prediction separately, and the authors stated that few studies have coupled the two models. But the authors did not include those few studies and the drawbacks or gaps. Thus, the reviewer suggests to mention the past studies that focused on coupling of SWAT and SVM, and the novel idea of the current study.

**Answer:** *We have added some literature review into the revision to summary and elaborate the pervious related research and highlighted the innovation of this study.* *The part of revision showed as follow:*

*“	Several works have evaluated the performance of SWAT and SVM in streamflow prediction [**12**,* *19**,* *28**].* *Zhang, Srinivasan [12]* *et al. applied Artificial Neutral Network (ANN) and SVM methods to identify the optimal SWAT parameters to save the time cost of calibration and improve the efficiency of parameter calibration in two watersheds of the U.S.* *Jajarmizadeh, Kakaei Lafdani [28]* *et al. compared the monthly streamflow predictions from SWAT and SVM, and found the SVM model has a closer value for the average flow in comparison to the SWAT model. These efforts, however, either applied SVM in searching the optimal calibration parameters or built separate SWAT and SVR models, then estimated their running performance. Few studies have combined the two methods for a hybrid approach to streamflow prediction.* *Chiogna, Marcolini [19]* *et al. developed an SVM with SWAT model to predict hydropeaking in alpine watersheds in the Northeastern Italian. They used SVM to train the output of SWAT and found the SVM model can capture the fluctuation in streamflow. To the best of author’s knowledge, no study has coupled the SVM and SWAT and considered wet-dry change for streamflow prediction. The objective of this study is to show how a support vector regression (SVR) method to support SWAT calibration can be used to improve monthly streamflow prediction for different seasons in the IRW.”*

Methodology:
2. On page 6, line 124 to 125, it was mentioned that multiple land use/soil/slope method was applied to define the HRUs in SWAT model with land use (10%), soil (10%) 125 and slope (5%) threshold. Is there any justification why these threshold values were selected?

**Answer:** *We appreciate reviewer’s concern and awareness on how to determine threshold of land use, soil, and slope. We described the process of dividing threshold (land use (10%), soil (10%) 125 and slope (5%) threshold) is subjective and based on experience. Unfortunately, there is not presently a unified guideline on how to determine these thresholds for SWAT model development, but more accepted practice than explicit rules. The upstream drainage area we extracted is very close with the results published by USGS, so we believe our method are reasonable and the current threshold approach is appropriate. Defining and study of the impact of different thresholds dividing methods on simulations of SWAT is incredibly interesting, but beyond the scope of our research.*

3. In this manuscript, it seems that SWAT-CUP calibration approach was used, and the modelled streamflow results are validated against measured ones. However, the authors did not mention the hydrologic parameters that control streamflow. When the authors discuss about the model performances, they compared SWAT-CUP against SWAT-SVR. But, it is difficult for the reader to understand easily how the model outcomes came especially for SWAT-CUP (example page 12, line 251 – 252). Moreover, the calibration and validation periods are not stated clearly,

***Answer:*** *We have appended the hydrologic parameters into the appendix part for reader’s understanding. We appreciate reviewer’s constructive comments. We need to emphasize that we used the spatial calibration method rather than temporal calibration method, and we applied the leave-one-out method to calibrate and validate the SWAT-SVR and SWAT-CUP. In each model run, the leave-one-out sampling method was applied to calibrate the SWAT-SVR model spatially. Out of n stations, one station was excluded for testing purposes, and the SWAT-SVR model was trained with the remaining (n-1) stations. This step was repeated until all stations had been removed once. SWAT-CUP passed through the same calibration process. We attached the calibration parameters and their range as below for reviewer’s reference.*

**Table 1. The initial parameters and their range in calibration.**

| No | Parameter Name^1^ | Parameter Description | Range | Season | |
| --- | --- | --- | --- | --- | --- |
|  |  |  |  | **If used in the wet season** | **If used in the dry season** |
| 1 | R__CN2.mgt | SCS runoff curve number II | -0.25-0.25 | Yes | Yes |
| 2 | V__ALPHA_BF.gw | Baseflow alpha factor (1 day^−1^) | 0–1 | Yes | Yes |
| 3 | V__GWQMN.gw | Threshold depth of water in the shallow aquifer required for return flow to occur (mm H_2_O) | 0–2000 | Yes | Yes |
| 4 | V__GW_REVAP.gw | Groundwater “revap” coefficient | 0.02–0.2 | Yes | Yes |
| 5 | V__EPCO.hru | Plant uptake compensation factor | 0–1 | Yes | Yes |
| 6 | R__SOL_K (1).sol | Saturated hydraulic conductivity at the 1st soil layer (mm h^−1^) | 30-102 | Yes | Yes |
| 7 | R__SOL_AWC (1).sol | Available water capacity of the 1st soil layer (mm H_2_O mm soil^−1^) | 0.08-0.2 | Yes | No |
| 8 | R__SOL_BD (1).sol | Moist bulk density at the 1st soil layer (g cm^−3^) | 1.3-1.45 | Yes | No |
| 9 | A__OV_N.hru | Manning’s “n” value for overland flow | 0.01–30 | Yes | No |
| 10 | A__CH_K2.rte | Effective hydraulic conductivity in main channel alluvium (mm h^−1^) | −0.01–500 | Yes | Yes |
| 11 | R__HRU_SLP.hru | Average slope steepness (m m^−1^) | 0–1 | Yes | Yes |
| 12 | V_RCHRG_DP.gw | Deep aquifer percolation fraction | 0-1 | Yes | Yes |
| 13 | A_CH_K1 | Effective hydraulic conductivity in tributary channel alluvium | 0-300 | Yes | No |
| 14 | V_ESCO.hru | Soil evaporation compensation factor | 0-1 | No | Yes |

^1^ Note: “*A*__”, “*V*__” and “*R*__” mean an absolute increase, a replacement, and a relative change to the initial parameter values, respectively.

4. One of the most important features of SWAT-CUP is its capability to determine the uncertainty level of SWAT model prediction using sequential uncertainty fitting 2 (SUFI-2) algorithm. However, the current study used SWAT-CUP- (SUFI-2) as a tool of calibration and validation method, the level of model uncertainties was missed or not explained sufficiently why it was not included.

**Answer:** *For SWAT-CUP, it is a relative mature calibration software. It can easily and automatically obtain 95PPU. Hence, we can get the P-factor and r-factor in an easy way. Discussing the uncertainty level of SWAT model prediction is not hard for SWAT-CUP. However, SWAT-SVR presently is a relatively new R script, we haven’t consider to calculate p-factor and r-factor and neither further focus on the uncertainty analysis and comparison of SWAT-SVR and SWAT-CUP output results, but that would be a good suggestion if our approach is adopted. We continue to improve the current scripts and develop more functions. We are considering ways to share our more innovative approaches in the future but this is beyond the scope of this manuscript.*

Result and discussions:
4. In the manuscript, it was mentioned that the predicted monthly streamflow by SWAT-SVR was more accurate during wet season than the dry season. Detail explanation is required why the model performances differ between the wet and dry seasons.

***Answer:*** *We have added line 366:*

*“Low flows took place in dry seasons are a seasonal phenomenon, and their prediction is a challenging task in hydrology [**58**]. This difficulty may be attributed to the complexity of groundwater processes and the lack of effective evaluation criteria of low flows. Low flows in the dry season are typically generated from groundwater discharge or surface discharge from lakes, reservoirs, and marshes [**58**]. However, it is hard to investigate subsurface water discharge from nearby watersheds into a river channel in an unclosed watershed because of the limitation of hydrological measurement methods and the complexity of groundwater flow processes. Often these types of groundwater models are highly site-specific [**59**] or cover vast areas [**60**]. Furthermore, there are no effective and suitable statistical indicators to estimate the performance of low flows simulation. Both R^2^ and NSE are known to put greater emphasis on high flows prediction and are sensitive to the hydrological regime, sample size or outliers [**61**].* *Pushpalatha, Perrin [61]* *suggested using the objective function NSE of SqrtQ or lnQ for low flows evaluation.”*

5. Page 12, line 251- 252, it was mentioned that “SWAT-CUP method also underestimated wet season streamflow but remarkably overestimated dry season streamflow. The SWAT-SVR model has approximately similar performances for the wet and dry seasons”. The reviewer believes that more discussions are required based on the feature of the two methods.

***Answer:*** *We have added extra analysis and explanation into the revision.*

*“Based on the value of PBIAS, the SWAT-SVR model slightly underestimated monthly streamflow for each watershed during the wet and dry seasons, and SWAT-CUP method also underestimated wet season streamflow but remarkably overestimated dry season streamflow. The average of NSE and R^2^ of 13 sites decreased from 0.92 and 0.92 in the wet season to -0.16 and 0.55 in the dry season, respectively. This results are consistent with* *Zhang, Chen [43]**’s study in which the SWAT model can produce good simulations for the wet season but poor simulations for the dry season. The possible reason is that R2 and NSE are sensitive to extremely large number (i.e. high flows took place in the wet season). Compared with the performance of SWAT-CUP, the SWAT-SVR model has approximately similar performances for the wet and dry seasons. Although the simulation results from SWAT-CUP had better overall performance (i.e. 53.8% of the runs had “Very Good” ratings) than those of the SWAT-SVR model in the wet season, SWAT-CUP failed to estimate monthly streamflow in the dry season, in which all runs were identified as “Unsatisfactory” ratings. We also noted that the variation of statistics is small, and the value of each indicator is close between different SWAT-SVR models. This is because a single SWAT-SVR watershed model was built based on eleven other common watersheds in calibration.”*

6. PLOS authors have the option to publish the peer review history of their article ([what does this mean?](https://gcc01.safelinks.protection.outlook.com/?url=https%3A%2F%2Fjournals.plos.org%2Fplosone%2Fs%2Feditorial-and-peer-review-process%23loc-peer-review-history&data=04%7C01%7CForshay.Ken%40epa.gov%7C4a4307e3f4a648dffa1508d8915b6184%7C88b378b367484867acf976aacbeca6a7%7C0%7C0%7C637419168959531451%7CUnknown%7CTWFpbGZsb3d8eyJWIjoiMC4wLjAwMDAiLCJQIjoiV2luMzIiLCJBTiI6Ik1haWwiLCJXVCI6Mn0%3D%7C2000&sdata=G%2F3r8f2eKJdQlfqNgY%2FzUJEDW3wZPThiAVEOB43R1n0%3D&reserved=0)). If published, this will include your full peer review and any attached files.

If you choose “no”, your identity will remain anonymous but your review may still be made public.

**Do you want your identity to be public for this peer review?** For information about this choice, including consent withdrawal, please see our [Privacy Policy](https://gcc01.safelinks.protection.outlook.com/?url=https%3A%2F%2Fwww.plos.org%2Fprivacy-policy&data=04%7C01%7CForshay.Ken%40epa.gov%7C4a4307e3f4a648dffa1508d8915b6184%7C88b378b367484867acf976aacbeca6a7%7C0%7C0%7C637419168959531451%7CUnknown%7CTWFpbGZsb3d8eyJWIjoiMC4wLjAwMDAiLCJQIjoiV2luMzIiLCJBTiI6Ik1haWwiLCJXVCI6Mn0%3D%7C2000&sdata=UcLd%2B5mWJWrzL8Twc6vtEmlY2Z0S6W%2BrbDDCQQe44ew%3D&reserved=0).

Reviewer #1: No

Reviewer #2: No

Reviewer #3: **Yes:** Tigabu, Tibebe B.
